# Outcomes after direct thrombectomy versus combined thrombolysis and thrombectomy in acute large artery occlusion stroke with atrial fibrillation

**DOI:** 10.3389/fneur.2026.1780191

**Published:** 2026-03-13

**Authors:** Hao Shu, He Huang, Xiaona Xu, Ruqian He

**Affiliations:** Department of Neurology, The Third Affiliated Hospital of Wenzhou Medical University (Ruian People’s Hospital), Wenzhou, Zhejiang, China

**Keywords:** atrial fibrillation, intravenous thrombolysis, outcomes, stroke, thrombectomy

## Abstract

**Background:**

The role of intravenous thrombolysis (IVT) before endovascular thrombectomy (EVT) remains controversial, particularly for patients with acute large vessel occlusion (LVO) due to atrial fibrillation (AF), who may have a poor response to thrombolysis. Furthermore, robust evidence is lacking regarding the benefits of bridging therapy in patients with AF-related AIS-LVO. Accordingly, this study aimed to assess whether patients with AF benefit from bridging thrombectomy.

**Methods:**

We performed a retrospective, observational, single-center study from January 2020 to June 2025. Patients meeting the inclusion criteria for both IVT and EVT were enrolled and dichotomized based on thrombectomy type: the bridging thrombectomy (IVT + EVT) group versus the direct thrombectomy (EVT alone) group. After 1:1 propensity score matching (PSM), the outcome measures, including the proportions of patients with modified Rankin scale (mRS) scores of 0–2 at 90 days, the number of retrieval attempts, successful recanalization, door-to-recanalization time, symptomatic intracranial hemorrhage, and mortality within 90 days, were compared. Finally, an exploratory subgroup analysis was performed, stratifying the cohort by age.

**Results:**

A total of 221 patients who underwent EVT were included (125 with bridging IVT and 96 with direct EVT). After PSM, there were no significant differences in 90-day functional independence (mRS 0–2) between the two groups (59.0% versus 50.0%; *p* = 0.158). Furthermore, direct EVT was associated with a shorter median door-to-recanalization time (125.5 versus 135.5 min; *p* = 0.015) and fewer median thrombectomy passes (1 versus 2, *p* = 0.003). The rates of successful recanalization, symptomatic intracranial hemorrhage, and 90-day mortality were comparable. A significant interaction effect between age and treatment modality was observed for the primary outcome of a 90-day mRS score of 0–2 (*p* for interaction = 0.048). Among patients aged ≥85 years, those receiving EVT alone had a significantly higher rate of functional independence than those in the combined IVT and EVT groups (50.0% versus 12.5%, *p* = 0.041).

**Conclusion:**

In this real-world, matched-control study, EVT alone demonstrated comparable efficacy to combined IVT + EVT for AF-related LVO. However, in patients aged ≥85, EVT alone significantly improved functional independence and reduced mortality.

## Introduction

Endovascular thrombectomy (EVT) has become the standard of care for patients with large vessel occlusion (LVO) (1). Despite guidelines recommending combined intravenous thrombolysis (IVT) and EVT for eligible patients (1, 2), the role of IVT before EVT remains controversial. Although robust evidence from randomized controlled trials (RCTs) (3–5) has established IVT plus EVT as standard therapy, DIRECT-MT and DEVT RCTs (6, 7) have also demonstrated the non-inferiority of direct EVT compared with IVT plus EVT. This outcome may be attributable to both the selected non-inferiority margins and to heterogeneity in the stroke population. Hence, individual patient characteristics and clinical variables, whether favoring direct or bridging thrombectomy, are crucial for determining the optimal reperfusion strategy. Atrial fibrillation (AF) is a prevalent cardiac arrhythmia and a major cause of LVO stroke, accounting for 25–40% of all cases (8). Therefore, it is of great clinical importance to investigate whether patients with AF-related LVO benefit from bridging thrombectomy.

Currently, the efficacy and safety of administering IVT prior to EVT for patients with AIS-LVO patients with AF who are directly admitted to thrombectomy-capable centers remain uncertain. Studies have demonstrated that patients with stroke and AF exhibit a poorer response to thrombolysis and a greater risk of intracerebral hemorrhage (ICH) than those without AF (9, 10). Histological studies have revealed that cardiac thrombi, which are predominantly composed of red blood cells with relatively low fibrin levels, are resistant to thrombolytic agents because of their distinct composition (11). Collectively, these factors may contribute to unfavorable clinical outcomes in patients with AF undergoing bridging thrombectomy. Current evidence regarding the optimal thrombectomy strategy for this population remains inconsistent. A *post-hoc* meta-analysis pooling individual patient data from SKIP and DEVT RCTs suggested that patients with AIS-LVO and AF may benefit from direct thrombectomy (12). Similarly, a *post-hoc* subgroup analysis of the DIRECT-MT RCT revealed that patients with mild and moderate cardioembolic stroke may benefit more from direct mechanical thrombectomy than from the bridging therapy (13). In contrast, several observational studies have suggested a potential benefit of bridging thrombectomy for patients with atrial fibrillation (14, 15). To date, however, no RCTs have specifically evaluated the effect of bridging thrombectomy in this subgroup.

Given the limited and conflicting evidence regarding the benefit of bridging thrombectomy specifically for patients with AIS-LVO complicated by AF, we aimed to determine whether patients with AF benefit from bridging thrombectomy.

## Methods

### Study design and population

This was a single-center, retrospective, observational study conducted from January 2020 to June 2025. The inclusion criteria were as follows: (1) Age ≥18 years; (2) diagnosis of AIS due to LVO, confirmed by computed tomography angiography (CTA) or digital subtraction angiography, involving either the anterior or posterior circulation; (3) National Institutes of Health Stroke Scale (NIHSS) score ≥6; (4) presence of atrial fibrillation; (5) eligibility for both IVT and EVT according to the American Heart Association/American Stroke Association guidelines (1); (6) underwent EVT. The exclusion criteria were as follows: (1) premorbid mRS score ≥2; (2) life expectancy of <3 months; (3) patients lost to follow-up.

As a real-world study reflecting routine clinical practice, the decision to administer IVT before EVT was made based on the physicians’ recommendations and patient or family preferences. Patients were divided into two groups according to whether IVT with rt-PA was administered before EVT: EVT alone and combined IVT + EVT. In the combined group, patients received intravenous rt-PA at a dose of 0.9 mg/kg (maximum 90 mg), administered as a 10% bolus followed by a continuous infusion of the remaining dose over 1 h. EVT was performed according to standard protocols in both groups. Informed consent was obtained from all the enrolled participants or their legal representative. Our study received approval from the Medical Ethics Committee of The Third Affiliated Hospital of Wenzhou Medical University (Approval Number: YJ2024012).

### Data collection

Clinical data were collected from the enrolled patients. The collected dataset included comprehensive baseline variables (demographics, medical history, admission NIHSS score, vital signs, laboratory results, and imaging parameters such as occlusion site and ASPECTS), procedural metrics (workflow times, number of thrombectomy passes, and mTICI scores), and outcome measures (90-day mRS score and rates of symptomatic/asymptomatic ICH). All imaging data, including baseline CTA and follow-up CT scans (obtained immediately post-MT, at 24 ± 2 h, and in case of neurological deterioration), were reviewed and evaluated by an independent imaging core laboratory.

### Outcome measures and definitions

The primary outcome was the proportion of patients who achieved a favorable functional outcome, defined as an mRS score of 0–2 at 90 days. The mRS was assessed by trained researchers via face-to-face or telephone interviews, using a standardized protocol. The secondary outcomes included the distribution of 90-day mRS scores, time intervals from door to puncture and door to recanalization, and the number of thrombectomy passes. Safety outcomes included all-cause mortality within 90 days, any ICH within 24 h, and symptomatic ICH (sICH). sICH was defined according to the European Co-Operative Acute Stroke Study-II criteria as any hemorrhagic transformation associated with an increase in the NIHSS score of ≥4 points (16). AF was diagnosed based on electrocardiography and/or 24-h Holter monitoring, encompassing both previously known and newly diagnosed cases of paroxysmal or persistent AF.

### Statistical analysis

Normally distributed continuous data are reported as means ± standard deviations and compared with the Student’s *t*-test; nonnormally distributed data are presented as medians (IQRs) and compared with the Mann–Whitney *U* test. Categorical variables, expressed as numbers (%), were compared using Pearson’s chi-square test or Fisher’s exact test when expected cell counts were <5, as appropriate. Propensity score matching (PSM) was performed to minimize baseline differences between the treatment groups. First, variables with significant baseline imbalances between the two groups (*p* < 0.05) were identified, and potential confounders, including age, sex, and the NIHSS score, were incorporated into the propensity model. To this end, 1:1 propensity score matching was subsequently performed using a caliper width of 0.1 to minimize residual confounding. Outcomes in the matched cohort were assessed using multivariable logistic regression, adjusting for age, sex, the NIHSS score, medical history, ASPECTS and onset-to-arrival time. Results are reported as odds ratios (ORs) and 95% confidence intervals (CIs). Finally, an exploratory subgroup analysis was performed, stratifying the cohort by age. Based on prior studies (17, 18), patients aged ≥85 years are commonly defined as the “oldest-old” population; accordingly, we categorized patients into those aged <85 years and those aged ≥85 years. The potential difference in the treatment effect across these age subgroups was evaluated by testing the interaction between the treatment group and the age subgroup using a logistic regression model. All statistical analyses were performed using the Statistical Package for the Social Sciences (version 27.0), with a *p* < 0.05 considered statistically significant.

## Results

**Figure 1:**
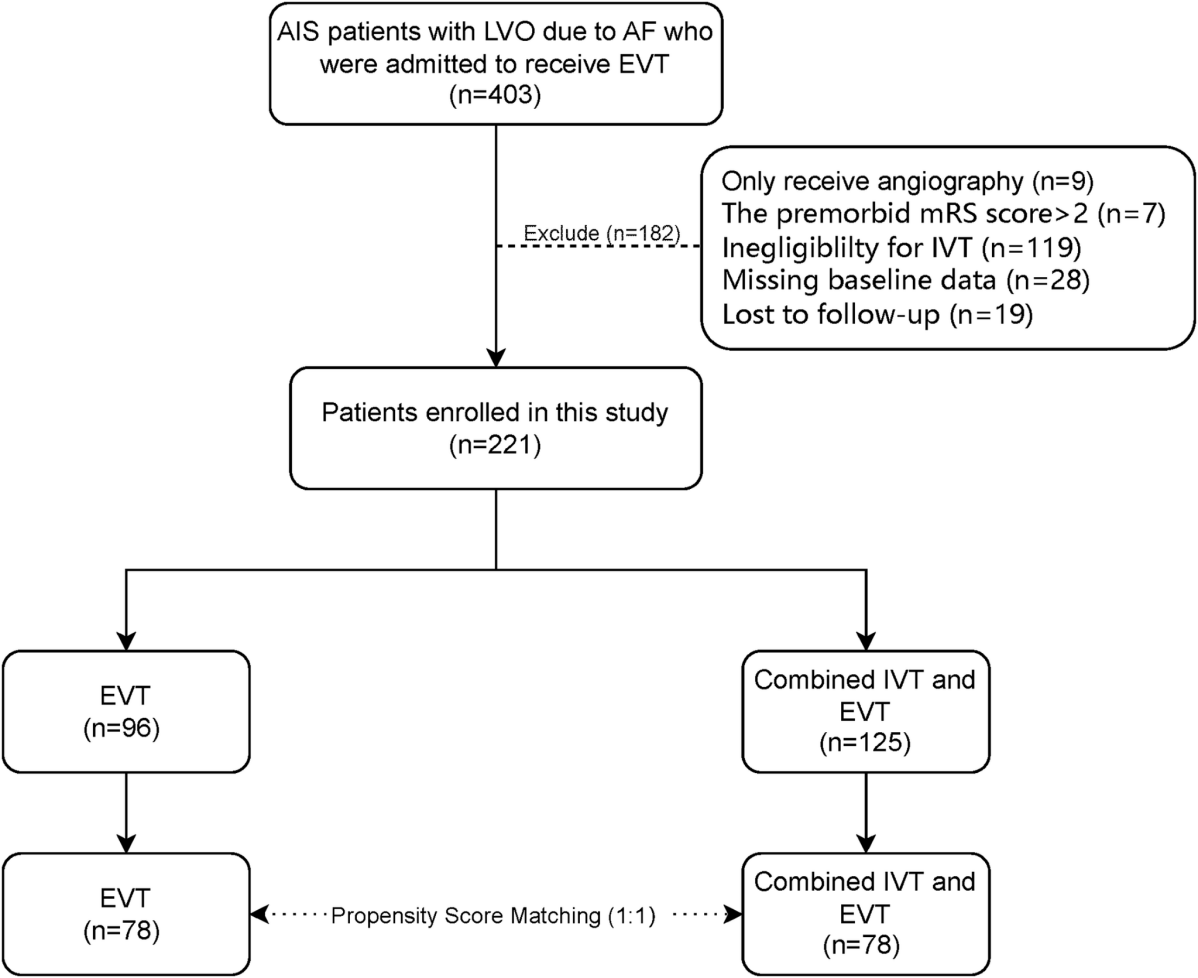
Flow chart study. AIS, acute ischemic stroke; AF, atrial fibrillation; EVT, endovascular thrombectomy; IVT, intravenous thrombolysis; LVO, large vessel occlusion; mRS, modified Rankin scale.

A total of 403 patients with AIS-LVO complicated by AF received EVT in our stroke center during the study period. Of these, 221 participants met the inclusion and exclusion criteria. The study enrollment flow is presented in Figure 1. These enrolled patients who underwent EVT, primarily with a stent retriever, aspiration, or a combination of both. Among them, 125 patients received bridging IVT, while the remaining patients proceeded directly to EVT, primarily due to refusal of consent by patients or their family members. Among the 221 patients, 127 (57.5%) were male, and the median age was 75 years (IQR, 68–82). A total of 204 patients (92.3%) had anterior circulation strokes. Successful recanalization (mTICI = 3) on the final angiogram was achieved in 172 patients (77.8%). A favorable functional outcome (mRS ≤2) was observed in 110 patients (49.8%), with a median score of 3 (IQR 1–5). sICH within 24 h occurred in 7 patients (3.2%). The overall mortality rate was 23.0% (51 patients).

### Baseline characteristics

The baseline characteristics of the study population are summarized in Table 1. Before PSM, significant differences in several baseline parameters were observed between the EVT alone group and the combined IVT and EVT group. Specifically, the EVT alone group presented with a significantly higher baseline NIHSS score (median: 18 versus 16; *p* = 0.031) and a higher incidence of prior ischemic stroke (18.8% versus 8.0%; *p* = 0.017). Other demographic characteristics, medical history, and most imaging findings were well balanced between the two cohorts at baseline. Following PSM, all baseline characteristics were well balanced, indicating successful matching.

**Table 1:** Baseline characteristics of patients with AF treated with EVT versus combined IVT and EVT.

Variables	Pre-matched population (*n* = 221)			Post-matched population (*n* = 156)		
	EVT	Combined IVT and EVT	*p*	EVT	Combined IVT and EVT	*p*
Demographic characteristics						
Age, year, median (IQR)	76.0 (68.2–82.0)	75.0 (67.0–83.0)	0.397	76.0 (68.0–82.0)	73.5 (65.8–83.0)	0.462
Male, *n* (%)	57 (59.4)	70 (56.0)	0.615	45 (57.7)	48 (61.5)	0.624
Medical history						
Hypertension, *n* (%)	62 (64.6)	74 (59.2)	0.415	50 (64.1)	51 (65.4)	0.867
Diabetes, *n* (%)	22 (22.9)	19 (15.2)	0.144	16 (20.5)	12 (15.4)	0.404
Coronary heart disease *n* (%)	12 (12.5)	7 (5.6)	0.070	8 (10.3)	6 (7.7)	0.575
Prior ischemic stroke, *n* (%)	18 (18.8)	10 (8.0)	0.017	9 (11.5)	8 (10.3)	0.797
Hyperlipidemia, *n* (%)	16 (16.7)	12 (9.6)	0.117	11 (14.1)	7 (9.0)	0.316
Smoking, *n* (%)	18 (18.8)	30 (24.0)	0.348	15 (19.2)	18 (23.1)	0.556
Alcohol consumption, *n* (%)	16 (16.7)	21 (16.8)	0.979	12 (15.4)	11 (14.1)	0.821
BMI, median (IQR)	23.0 (21.2–25.9)	23.15 (20.8–25.0)	0.487	23.3 (20.8–26.0)	22.3 (20.4–25.0)	0.241
Imaging characteristics						
Occlusion site on CT angiography, *n* (%)			0.731			0.908
ICA	29 (30.2)	30 (24.0)		22 (28.2)	19 (24.4)	
MCA	58 (60.4)	83 (66.4)		48 (61.5)	52 (66.7)	
ACA	2 (2.1)	2 (1.6)		1 (1.3)	1 (1.3)	
BA	7 (7.3)	10 (8.0)		7 (9.0)	6 (7.7)	
ASPECTS, median (IQR)	10 (8–10)	10 (9–10)	0.246	10 (9–10)	10 (9–10)	0.732
ACI, *n* (%)	89 (92.7)	115 (92.0)	0.845	71 (91.0)	72 (92.3)	0.772
Clinical examination at arrival						
NIHSS score, median (IQR)	18.0 (14.0–22.8)	16.0 (12.0–21.0)	0.031	15.0 (13.0–21.0)	18.0 (14.0–22.2)	0.382
Systolic blood pressure, mmHg, median (IQR)	152.0 (138.0–162.8)	148.0 (135.0–160.0)	0.601	151.5 (135.0–160.5)	147.0 (133.8–159.2)	0.725
Diastolic blood pressure, mmHg, median (IQR)	78.0 (73.0–89.0)	78.0 (70.0–85.5)	0.566	78.0 (70.0–87.2)	77.0 (68.8–85.0)	0.320
Glucose level, mmol/L, median (IQR)	7.1 (6.2–9.1)	7.3 (6.1–9.0)	0.751	7.1 (6.3–9.1)	7.6 (6.0–9.1)	0.990
Workflow times						
Onset to arrival, min, median (IQR)	76.0 (48.0–123.0)	65.0 (46.5–102.0)	0.169	75.0 (46.5–121.0)	65.5 (45.8–101.0)	0.385

Statistically significant (*p* ≤ 0.05).

AF, atrial fibrillation; EVT, endovascular thrombectomy; IVT, intravenous thrombolysis; IQR, interquartile range; BMI, body mass index; NIHSS, National Institutes of Health Stroke Scale; ASPECTS, Alberta Stroke Program Early CT Score; ACI, anterior circulation infarction; CT, computed tomography; ICA, internal carotid artery; MCA, middle cerebral artery; ACA, anterior cerebral artery; BA, basilar artery.

### Outcome measures

**Figure 2:**
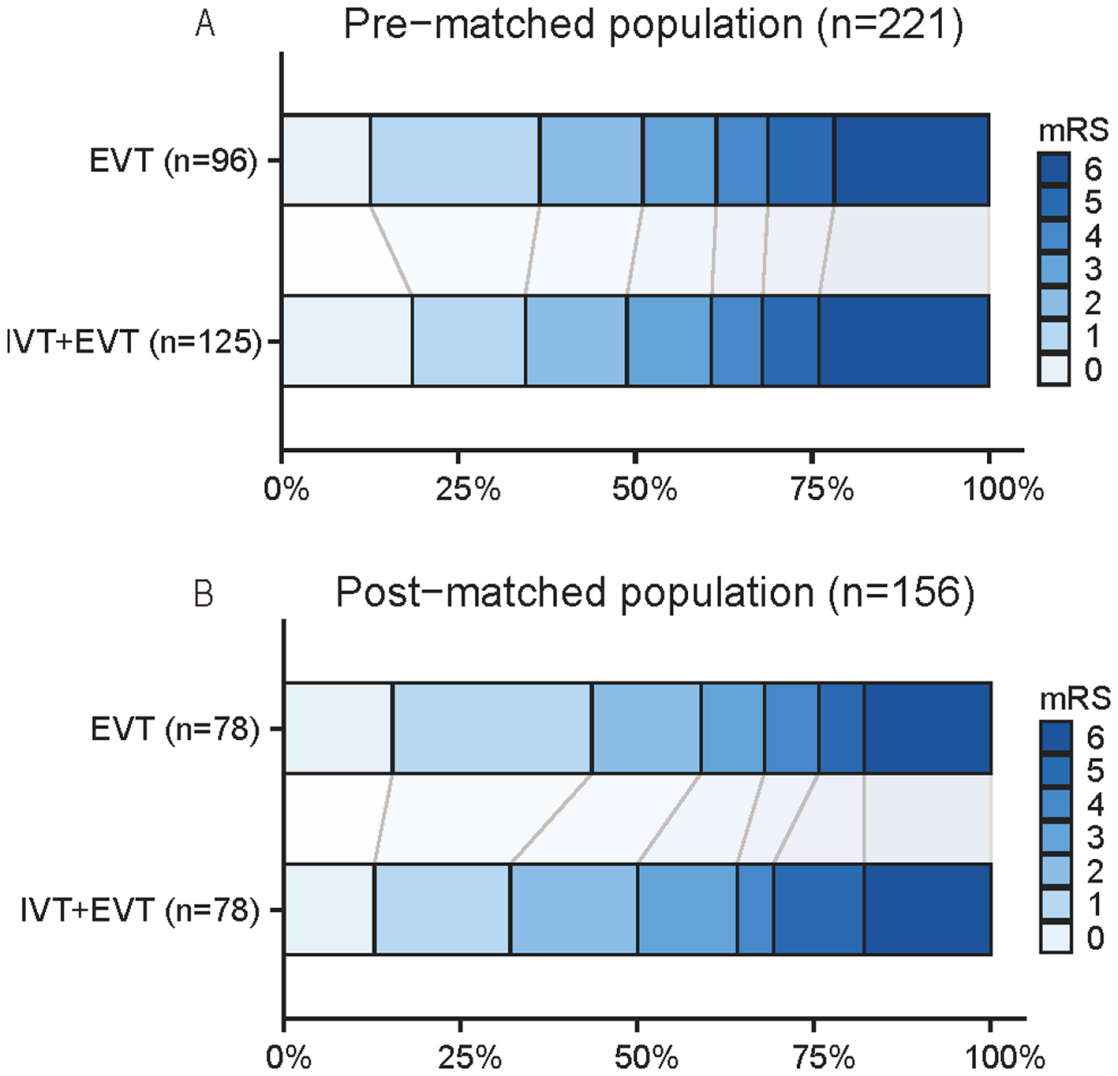
The 90-day modified Rankin scale (mRS) distributions for bridging therapy (IVT + EVT) versus direct EVT, before **(A)** and after **(B)** propensity score matching. EVT, endovascular thrombectomy; IVT, intravenous thrombolysis.

A comparison of the outcome measures between the two groups after PSM is presented in Table 2. Logistic regression analysis of the primary outcome—the proportion of patients with an mRS score of 0–2 at 90 days—revealed no significant difference between the two groups in the matched cohort [59.0% versus 50.0%; OR, 0.59 (95% CI, 0.29–1.22); *p* = 0.158]. Regarding secondary outcomes, patients who underwent EVT alone had significantly shorter door-to-puncture times [median, 97.0 versus 101.0 min; OR, 16.64 (95% CI, 3.81–29.46); *p* = 0.012] and door-to-recanalization times [median, 125.5 versus 135.5 min; OR, 18.90 (95% CI, 3.80–34.00); *p* = 0.015] and required fewer thrombectomy passes [median, 1 versus 2; OR, 2.62 (95% CI, 1.34–5.14); *p* = 0.003] than did those in the combined IVT and EVT group. However, no significant differences in other secondary outcomes, including the median mRS score at 90 days [2.0 versus 2.5; common OR, 1.44 (95% CI, 0.81–2.56); *p* = 0.107], or the rate of successful recanalization [mTICI = 3, 75.6% versus 80.8%; OR, 1.42 (95% CI, 0.65–3.09); *p* = 0.380], were detected between the two groups. With respect to safety outcomes, the incidence of symptomatic intracerebral hemorrhage within 24 h [3.8% versus 1.3%; OR, 0.47 (95% CI, 0.04–5.16); *p* = 0.533], death within 90 days [17.9% versus 17.9%; OR, 1.00 (95% CI, 0.40–2.50); *p* = 0.998], and asymptomatic hemorrhage [17.9% versus 24.4%; OR, 1.81 (95% CI, 0.78–4.16); *p* = 0.164] did not differ significantly between the EVT alone and combined therapy groups. The distribution of the 90-day mRS scores is illustrated in Figure 2.

**Table 2:** Outcome measures of AF patients treated with EVT versus combined IVT and EVT after 1:1 propensity score matching.

Variables	Total (*N* = 156)	EVT	Combined IVT and EVT	OR (95% CI)	*p*-value
Primary outcomes					
mRS 0–2 at 90 days, *n* (%)	85 (54.5)	46 (59.0)	39 (50.0)	0.59 (0.29–1.22)	0.158
Secondary outcomes					
mRS at 90 days, median (IQR)	2.0 (1.0–5.0)	2.0 (1.0–4.2)	2.5 (1.0–5.0)	1.44 (0.81–2.56)	0.107
Door to puncture, min, median (IQR)	98.5 (83.2–121.8)	97.0 (80.0–116.5)	101.0 (87.2–130.5)	16.64 (3.81–29.46)	0.012^a^
Door to recanalization, min, median (IQR)	129.5 (109.8–163.0)	125.5 (107.8–151.5)	135.5 (115.0–174.8)	18.90 (3.80–34.00)	0.015^a^
Pass number of thrombectomy, median (IQR)	1 (1.0–2.0)	1 (1.0–2.0)	2 (1.0–2.0)	2.62 (1.34–5.14)	0.003^a^
mTICI = 3, *n* (%)	122 (78.2)	59 (75.6)	63 (80.8)	1.42 (0.65–3.09)	0.380
Safety outcomes					
Death within 90 days, *n* (%)	28 (17.9)	14 (17.9)	14 (17.9)	1.00 (0.40–2.50)	0.998
Symptomatic ICH within 24 h, *n* (%)	4 (2.6)	3 (3.8)	1 (1.3)	0.47 (0.04–5.16)	0.533
Asymptomatic ICH *n* (%)	33 (21.2)	14 (17.9)	19 (24.4)	1.81 (0.78–4.16)	0.164

AF, atrial fibrillation; EVT, endovascular treatment; IVT, intravenous thrombolysis; mRS, modified Rankin scale; OR, odds ratio; CI, confidence interval; IQR, interquartile range; mTICI, modified thrombolysis in cerebral infarction scale; ICH, intracranial hemorrhage.

a Statistically significant (*p* ≤ 0.05).

### Subgroup analysis

Subgroup analyses stratified by patient age (<85 versus ≥85 years) are summarized in Table 3. A significant interaction effect between age and treatment modality was observed for the primary outcome of a 90-day mRS score of 0–2 (*p* for interaction = 0.048). Among patients aged ≥85 years, those receiving EVT alone had a significantly greater rate of functional independence than did those in the combined IVT and EVT groups [50.0% versus 12.5%; OR, 0.14 (95% CI, 0.02–0.92); *p* = 0.041]. In contrast, no significant difference was found in the <85 years subgroup [60.6% versus 59.7%; OR, 0.96 (95% CI, 0.47–1.95); *p* = 0.915]. Similar age-related effects were observed for the other key outcomes. For the median 90-day mRS score, a significant interaction was present (*p* for interaction = 0.023), with older patients (aged ≥85 years) in the EVT alone group demonstrating more favorable outcomes [median 2.5 versus 6; OR, 5.05 (95% CI, 1.20–21.34); *p* = 0.019], whereas no difference was observed in the younger subgroup. Moreover, a significant interaction was detected for 90-day mortality (*p* for interaction = 0.006). In patients ≥85 years, the EVT-alone group had a significantly lower mortality rate [16.7% versus 56.3%; OR, 6.43 (95% CI, 1.05–39.33); *p* = 0.044], whereas no significant between-group difference was observed in the <85 years subgroup. No significant interaction effects were identified for other secondary or safety outcomes, including successful recanalization (mTICI = 3), sICH, or asymptomatic hemorrhage (all *p*-values for interaction >0.05).

**Table 3:** Outcomes of EVT versus combined IVT + EVT by age subgroup.

Subgroup	EVT (*n* = 78)	Combined IVT and EVT (*n* = 78)	OR (95% CI)	*p*-value	*p* for interaction
Primary outcomes					
mRS 0–2 at 90 days, *n* (%)					
≥85 years	6/12 (50)	2/16 (12.5)	0.14 (0.02–0.92)	0.041^a^	0.048^a^
<85 years	40/66 (60.6)	37/62 (59.7)	0.96 (0.47–1.95)	0.915	
Secondary outcomes					
mRS at 90 days, median (IQR)					
≥85 years	2.5 (0.2–4.8)	6.0 (3.0–6.0)	5.05 (1.20–21.34)	0.019^a^	0.023^a^
<85 years	2.0 (1.0–4.2)	2.0 (1.0–4.0)	1.02 (0.55–1.88)	0.475	
Safety outcomes					
Mortality within 90 days, *n* (%)					
≥85 years	2/12 (16.7)	9/16 (56.3)	6.43 (1.05–39.33)	0.044^a^	0.006^a^
<85 years	12/66 (18.2)	5/62 (8.1)	0.40 (0.13–1.20)	0.100	
Symptomatic ICH within 24 h, *n* (%)					
≥85 years	1/12 (8.3)	1/16 (6.3)	0.73 (0.04–13.05)	0.833	0.242
<85 years	2/66 (3.0)	0/62 (0.0)	—^b^	0.996	
Asymptomatic ICH, *n* (%)					
≥85 years	3/12 (25.0)	3/16 (18.8)	0.69 (0.11–4.24)	0.691	0.370
<85 years	11/66 (16.7)	16/62 (25.8)	1.74 (0.74–4.12)	0.208	

EVT, endovascular treatment; IVT, intravenous thrombolysis; mRS, modified Rankin scale; OR, odds ratio; CI, confidence interval; IQR, interquartile range; ICH, intracranial hemorrhage.

a Statistically significant (*p* ≤ 0.05).

b Not estimable due to zero event count in one group.

## Discussion

In patients with AF-related LVO, direct EVT is at least as effective as combined IVT + EVT for the 90-day mRS outcome, consistent with the findings of DIRECT-MT and DEVT trials (6, 7) and a meta-analysis of observational studies among IVT-eligible patients (19). More specifically, among patients with AF, our findings are also consistent with two *post-hoc* analyses and a meta-analysis focusing on this subgroup, which indicated that direct EVT was noninferior to bridging therapy and even suggested a potential benefit of the direct EVT approach in this patient population (12, 13, 20).

Current guidelines recommend administering bridging thrombectomy within 4.5 h of stroke onset for patients with AIS-LVO who are eligible for both IVT and EVT, regardless of whether they have atrial fibrillation or not (1, 21). Furthermore, although AF is associated with a poorer response to thrombolysis, two studies demonstrated that the presence of AF did not significantly influence the treatment effect of bridging IVT compared with that in patients with no AF (22, 23). Nevertheless, our study demonstrated that direct EVT achieved comparable efficacy to the combined IVT + EVT approach among patients with AF. Several interrelated factors may explain this finding. First, in this study, direct EVT was associated with significantly shorter door-to-puncture times than those observed with combined IVT + EVT, which are known to contribute to improved clinical outcomes (24). Our real-world data, consistent with the ANGEL-ACT registry (25), demonstrate that the longer door-to-puncture time delay in the IVT + EVT group may partially the potential benefits of IVT. This prolonged delay likely reflects clinical realities, such as the need to complete IVT infusion, provide patient or family counseling, prepare the drug, and perform post-thrombolysis assessment. Second, our study revealed that patients who received EVT alone had a significantly shorter median door-to-recanalization times than did those in the combined IVT + EVT group (125.5 versus 135.5 min). Earlier recanalization is strongly associated with improved clinical outcomes in patients with acute ischemic stroke (26). Our findings suggest that the prolonged time associated with bridging IVT may partially offset its theoretical benefits. Finally, our study revealed a greater number of thrombectomy passes in the IVT + EVT group than in the direct EVT group. This observation, which aligns with previous studies (25), may be explained by intraprocedural thrombus fragmentation. IVT may promote thrombus fragmentation, leading to thrombus migration from proximal to distal vessels and thereby increasing retrieval difficulty (27). Notably, this study specifically investigated patients with AF. In the context of cardioembolic stroke, previous studies have demonstrated that IVT may also lyse thrombi within the left atrial appendage, potentially precipitating new thromboembolic events (28).

Furthermore, intravenous r-tPA has been demonstrated to cause greater vessel injury and blood–brain barrier disruption than EVT alone does, a phenomenon that may explain the elevated risk of ICH observed after IVT, which is particularly pronounced in Asian patients (29, 30). A recent study revealed that, compared with patients without AF, those with AF who received bridging therapy had a significantly greater risk of symptomatic intracranial hemorrhage and parenchymal hematoma type 2, without any improvement in 90-day functional outcomes (31). However, our study revealed no statistically significant difference in symptomatic hemorrhage rate between direct EVT and IVT + EVT in AF patients, with the direct EVT group even demonstrating a numerically higher rate. This evidence appears to confirm the safety of IVT plus EVT. However, this outcome is primarily due to the small sample size and the low incidence of symptomatic hemorrhage, both of which introduce bias and undermine its clinical relevance. Therefore, future studies with larger sample sizes are needed.

We performed an exploratory subgroup analysis stratified by age, revealing that in patients with AF aged >85 years, direct thrombectomy was associated with better clinical outcomes than bridging therapy. To date, there is limited evidence regarding which specific subgroups of patients with AF are most likely to benefit from bridging EVT. The ANGEL-ACT registry study, which included strokes of all etiologies, revealed no significant differences in the age-stratified subgroup analysis (comparing patients aged <65 versus ≥65 years) (25). Our primary rationale for stratifying by age was the objective nature of this variable, which is not subject to bias and is highly feasible in clinical practice. However, our subgroup analysis of patients aged >85 years was limited by a small sample size; therefore, the results should be interpreted with caution. To address this, future studies should use larger cohorts to further investigate the role of age stratification.

The novelty of this study lies in its subgroup analysis of AF patients within the ongoing debate over direct versus bridging thrombectomy, with subsequent age stratification enabling more personalized clinical decision-making. However, several limitations should be addressed. First, as a single-center observational study, this work is susceptible to selection bias, including that introduced by variations in physician’s clinical decision-making regarding IVT administration. Second, despite propensity score matching, residual confounding from both measured and unmeasured factors may persist, unlike in a randomized controlled trial. Moreover, the matching process further reduced the sample size, resulting in a limited cohort. Therefore, future studies with larger sample sizes are warranted to validate these findings. Third, in the age-based subgroup analysis of patients with AF, the limited sample size resulted in low statistical power. Consequently, the results can only indicate a potential trend, and considerable caution must be exercised in their interpretation. Fourth, we did not document the incidence of intraprocedural thrombus fragmentation, which is a factor that could affect functional outcomes. Finally, we did not include a detailed analysis of the impact of anticoagulants.

## Conclusion

In this real-world, matched-control study, EVT alone demonstrated efficacy comparable to combined IVT + EVT for AF-related LVO. However, in patients aged ≥85 years, EVT alone significantly improved functional independence and reduced mortality.

## Data Availability

The original contributions presented in the study are included in the article/supplementary material, further inquiries can be directed to the corresponding author.
